# The Royal Infirmary, Glasgow

**Published:** 1905-03-18

**Authors:** 


### Editor's Letter-Box.

### THE ROYAL INFIRMARY, GLASGOW.

Mr. H. C. Clark, C.M.G., Senior Surgeon, writes:—Your issue of Saturday last contains a leading article on "Hospital Control and Efficiency," in the course of which you make reference to the Glasgow Royal Infirmary in terms which seem to me to be likely to convey a wrong impression. It is true that part of the hospital building is more than a hundred years old and another part fifty years old, and also that the complete rebuilding of the Infirmary is about to be entered on; but it is not the case that the managers have been less active in carrying out continuous improvement than the managers of the Western Infirmary whose action you so commend. To mention only two out of a multitude of instances, I may state that the single operating theatre has been replaced by six theatres furnished and fitted with all modern requirements, and that the selection, education, and training of the large nursing staff is in advance of that in any other hospital in the kingdom. Neither in regard to efficiency nor economy does the Glasgow Royal Infirmary fear comparison with other general hospitals. Of the three Glasgow hospitals it stands second as regards its general mortality and is lowest in its operation mortality; while its cost per bed is several pounds below that of the two other similar institutions.

.˙. We did not intend to reflect upon the managers who under very great difficulties have done all that has been possible to make the best of old and faultily-constructed buildings. Our point was and is that no useful purpose can be served by a comparison between a hospital about to be razed to the ground and one like the London, where some £400,000 is being spent in making it replete with every modern improvement. When the Glasgow Royal Infirmary has been rebuilt comparisons may be equitably and profitably made. Mrs. Strong deserves the greatest credit for having originated the system of a preliminary school for intending probationers, an example followed by the London in the Tredegar House scheme and now generally adopted.

Ed. The Hospital.

